# A high high-density lipoprotein level is associated with Gleason score upgrade in Chinese patients diagnosed with high-grade prostate carcinoma

**DOI:** 10.1186/s12894-022-01155-9

**Published:** 2023-01-10

**Authors:** Jingcheng Lyu, Lu Li, Tianyu Jiang, Zhipeng Wang, Yichen Zhu

**Affiliations:** 1 Department of Urology, Capital Medical University Beijing Friendship Hospital, 95 Yong’an Road, Xicheng District, Beijing, 100050 China; 2 Department of Anesthesiology, Capital Medical University Beijing Friendship Hospital, 95 Yong’an Road, Xicheng District, Beijing, 100050 China

**Keywords:** Gleason score upgrading, Prostate cancer, Prostate biopsy, Radical prostatectomy

## Abstract

**Background:**

The high incidence of Gleason score upgrading (GSU) made urologists underestimate the disease, leading to the inaccurate therapeutic decision. The study aimed to explore relevant laboratory examination evidence associated with GSU.

**Methods:**

Patients diagnosed with prostate carcinoma undergoing radical prostatectomy in our center between January 2015 and December 2019 were included in this retrospective study. Patients were divided into GSU and NGSU groups according to the occurrence of GSU. Medical records were reviewed and analyzed between groups.

**Results:**

A total of 130 patients were enrolled, including 52 patients diagnosed with GS = 6 (20 NGSU and 32 GSU) and 78 patients with GS = 7 (36 NGSU and 42 GSU). No significant differences in demographic characteristics were found between groups. An increased neutrophil count (OR = 1.326, 95% CI = 1.005–1.748) and a decreased percentage of lymphocytes (OR = 0.951, 95% CI = 0.904–1) were associated with GSU in the GS = 6 group, whereas a high HDL level (OR = 7.735, 95% CI = 0.998–59.957) was associated with GSU in GS = 7 group. Preoperative high neutrophile count and low lymphocyte percentage were correlated with GSU in patients with low-grade prostate cancer. In contrast, high HDL level was associated with GSU in patients with high-grade prostate cancer.

**Conclusions:**

These laboratory examination data could provide urologists with information before making a therapeutic protocol.

## Background

Prostate cancer is a common urologic carcinoma and a usual cause of cancer deaths in men. Gleason score (GS) is the most used histologic grading system to score the invasiveness of prostate cancer, which guides the clinical therapeutic modalities and prognosis [1]. However, many studies showed that patients often have higher histological GS at prostatectomy than preoperative biopsy [1–5], and the occurrence of Gleason score upgrading (GSU) was around 50% [2, 3, 6–8] of all cases. The inaccuracy of prostate biopsy GS could make physicians underestimate the disease and make treatment decisions inaccurately. Therefore, identifying relevant biochemical or biological markers for GSU prediction is needed for better stratification of risk groups.

Previous studies showed an association between serum cholesterol levels and prostate cancer severity. A clinical study showed that patients with high-grade prostate cancer also tended to have high serum high-density lipoprotein (HDL) levels [9, 10]. A Finnish randomized study of 17-year median follow-up showed that high HDL might increase the risk of overall prostate cancer [11]. However, whether HDL is a GSU risk factor remains unknown.

Inflammatory response factors play a role in cancer progression, and many are used as indicators of the inflammatory status of patients with carcinoma. Some studies reported that neutrophile to lymphocyte ratio (NLR) ≥ of 2.5–3 was highly correlated with GSU in patients with low-risk prostate cancer [12–14]. Therefore, this study aimed to explore relevant laboratory examination evidence such as HDL and Inflammatory response factors associated with GSU in prostate cancer patients.

## Methods

### Study design

This study examined patients with prostate carcinoma who underwent radical prostatectomy at our center between January 2015 and December 2019. The Institutional Review Board of Capital Medical University Beijing Friendship Hospital approved this study (Approval number: 2020-P2-034-01). Additionally, informed written consent was obtained from all patients in this study. All procedures followed applicable guidelines and regulations. This retrospective study included patients diagnosed with prostate carcinoma via prostate biopsy. Exclusion criteria were: (1) History of previous surgery or trauma to the lower urinary tract; (2) History of any other carcinoma; (3) History of alpha-reductase inhibitor use; (4) incomplete medical records, and (5) Patients with a time interval between biopsy and RP of more than 8 months were also excluded from this study to rule out prostate cancer growing and upgrading and to cause GSU. All information was obtained from medical records. The included patients who met the criteria were divided into GSU and NSGU groups based on the occurrence of GSU.

All patients underwent transrectal ultrasound-guided prostate biopsy and multiparametric magnetic resonance imaging to diagnose prostate cancer using the Prostate Imaging-Reporting and Data System version 2 guidelines (PI-RADS v2). All the patients in both NGSU and GSU groups were first-time biopsy. The disposable core biopsy instruments were produced by Bard Peripheral Vascular, Inc in the U.S.A. The model number was MC1825 and C1816B, 18G, the sample notch length was 1.8 cm, and penetration depth was 22 mm. The mean number of biopsy cores of the NGSU group was 23.71 ± 2.14, ranging from 12 to 27 cores. Meanwhile, the mean number of biopsy cores in the GSU group was 23.24 ± 3.26, the same as the NGSU group, but ranged from 12 to 30. The prostate volume was computed. Transrectal ultrasound was used to determine the prostate volume (PV). GS was calculated using the modified Gleason scoring system developed by the International Society of Urological Pathology in 2005. Each patient's prostate-specific antigen (PSA) level was determined prior to prostate manipulation. The percentage of lymphocytes was calculated by dividing the number of lymphocytes by the total number of white blood cells. Between groups, variables such as age, PSA levels, PV, number of biopsy cores, percentage of biopsy tumors in non-peripheral areas, PI-RADS grading, metabolic syndrome, clinical stage, and blood examination data were compared.

### Statistical analysis

To detect differences between groups, baseline categorical variables were compared using Chi-square or Fisher's exact tests. The data distributions for continuous variables were compared using independent t-tests. Data were expressed as number (percentage) and, for continuous variables, as mean ± SD. We used multivariate logistic regression models to estimate the odds ratio (OR) and 95% confidence intervals (CIs). To develop a model predicting the outcomes, we used backward selection to enter variables with *p*-value < 0.15 in Table 1 into the model and retain variables with *p*-value < 0.05. A two-sided *P*-value of < 0.05 was regarded as statistically significant. Data management and statistical analyses were conducted using SAS version 9.4 (SAS Institute, Inc.).

**Table 1:** Characteristic of population according to GSU and NGSU group

Term	GS = 6			GS = 7		
	NGSU	GSU	*P*-value	NGSU	GSU	*P*-value
Case	20 (38.5%)	32 (61.5%)		36 (46.2%)	42 (53.8%)	
Age	65.60 ± 5.48	65.94 ± 5.71	0.833	67.03 ± 7.03	66.43 ± 5.71	0.684
BMI	24.27 ± 3.07	25.36 ± 2.91	0.207	24.82 ± 3.18	24.61 ± 3.17	0.775
Hypertension	6 (30.0%)	14 (70.0%)	0.389	14 (42.4%)	19 (57.6%)	0.649
Diabetes Mellitus	4 (33.3%)	8 (66.7%)	0.747	8 (53.3%)	7 (46.7%)	0.576
MetS	4 (28.6%)	10 (71.4%)	0.524	10 (41.7%)	14 (58.3%)	0.631
Total-PSA (ng/ml)	10.91 ± 7.97	12.71 ± 14.87	0.621	23.56 ± 27.47	26.16 ± 22.71	0.648
Free-PSA (ng/ml)	1.37 ± 0.90	2.05 ± 3.40	0.424	1.64 ± 1.09	2.29 ± 2.41	0.172
PSA			0.999			0.245
PSA ≤ 4	2 (33.3%)	4 (66.7%)		0 (0.0%)	3 (100.0%)	
PSA > 4	18 (39.1%)	28 (60.9%)		36 (48.0%)	39 (52.0%)	
Free PSA/Total PSA ratio			0.739			0.662
≤ 18%	13 (39.4%)	20 (60.6%)		27 (46.6%)	31 (53.4%)	
> 18%	4 (30.8%)	9 (69.2%)		3 (60.0%)	2 (40.0%)	
PV (ml)	82.07 ± 42.47	83.68 ± 41.95	0.907	88.77 ± 51.57	73.99 ± 29.62	0.205
PI-RADS			0.185			0.126
1	1 (50.0%)	1 (50.0%)		0 (0.0%)	1 (100.0%)	
2	7 (70.0%)	3 (30.0%)		8 (57.1%)	6 (42.9%)	
3	2 (22.2%)	7 (77.8%)		3 (30.0%)	7 (70.0%)	
4	9 (34.6%)	17 (65.4%)		18 (60.0%)	12 (40.0%)	
5	1 (20.0%)	4 (80.0%)		7 (30.4%)	16 (69.6%)	
Number of biopsy cores	24.10 ± 0.45	24.16 ± 0.52	0.689	23.33 ± 2.65	22.62 ± 4.20	0.381
Rate of positive cores in biopsy	0.18 ± 0.13	0.21 ± 0.17	0.384	0.29 ± 0.19	0.38 ± 0.22	0.066
Clinical stage			0.576			0.345
T1	2 (66.7%)	1 (33.3%)		2 (66.7%)	1 (33.3%)	
T2	16 (37.2%)	27 (62.8%)		30 (47.6%)	33 (52.4%)	
T3	2 (33.3%)	4 (66.7%)		3 (27.3%)	8 (72.7%)	
Triglyceride (mg/dL)	1.79 ± 1.40	1.65 ± 1.95	0.791	1.37 ± 0.87	1.44 ± 0.92	0.773
HDL-C (mg/dL)	1.13 ± 0.16	1.17 ± 0.40	0.658	1.08 ± 0.20	1.20 ± 0.28	**0.044**
Lymphocyte counts (× 10^9^/L)	1.58 ± 0.52	1.46 ± 0.82	0.563	1.42 ± 0.86	1.55 ± 0.71	0.441
Percentage of lymphocyte (%)	26.44 ± 9.02	19.56 ± 13.26	**0.031**	20.63 ± 12.74	19.85 ± 12.04	0.783
Neutrophile counts (× 10^9^/L)	4.13 ± 1.69	5.80 ± 3.13	**0.016**	5.69 ± 3.42	6.84 ± 3.22	0.133
Percentage of neutrophile (%)	64.03 ± 11.94	69.42 ± 18.24	0.204	71.91 ± 14.56	69.10 ± 17.91	0.454
NLR	3.38 ± 3.50	6.84 ± 7.61	**0.031**	6.77 ± 8.29	8.46 ± 14.11	0.530
Hb (g/L)	137.65 ± 9.70	130.31 ± 17.46	0.057	132.39 ± 14.64	128.12 ± 17.40	0.249

Significant value was in bold

*GSU* Gleason score upgrading, *PSA* prostate-specific antigen, *PV* prostate volume, *PI-RADS* prostate imaging reporting and data system, *HDL-C* high-density lipoprotein cholesterol, *Hb* hemoglobin, *ISUP* International Society of Urological Pathology

^a^Fisher’s exact test

## Results

A total of 130 patients were included in this study, with 52 patients diagnosed with GS = 6 and 78 patients with GS = 7. Patients’ baseline characteristics and clinical information are summarized in Table 1. In the GS = 6 group, 32 (61.5%) patients were with GSU. The mean age were 65.6 ± 5.48 and 65.94 ± 5.71 (*P* = 0.833) years in NGSU and GSU groups, with the percentage of lymphocytes of 26.44 ± 9.02% and 19.56 ± 13.26% (*P* = 0.031), neutrophile counts of 4.13 ± 1.69 and 5.8 ± 3.13 (*P* = 0.016), respectively. In the GS = 7 group, 42 (53.8%) patients were with GSU. The mean age were 67.03 ± 7.03 and 66.43 ± 5.71 (*P* = 0.684) years in NGSU and GSU groups, with serum HDL levels of 1.08 ± 0.2 and 1.2 ± 0.28 (*P* = 0.044), respectively. No statistically significant differences were displayed in age, BMI, hypertension, diabetes mellitus, metabolic syndrome, PSA levels, PV, number of biopsy cores, clinical stage, lymphocyte counts, and percentage of neutrophile between NGSU and GSU groups in either GS = 6 or GS = 7 group.

Univariable analysis was performed in both groups for further clarification (Table 2). In GS = 6 group, the percentage of lymphocyte (OR: 0.951, 95% CI: 0.904–1.000) and neutrophile count (OR: 1.326, 95% CI: 1.005–1.748) have significant effects on GSU. In the GS = 7 group, high HDL-C has a higher risk of GSU (OR: 7.735, 95% CI: 0.998–59.957) (Table 2).

**Table 2:** Univariate analysis

Term	GS = 6, GSU (reference = NGSU)			GS = 7, GSU (reference = NGSU)		
	OR	95% CI	*P* value	OR	95% CI	*P* value
Age	1.011	0.914–1.119	0.830	0.985	0.917–1.058	0.675
BMI	1.138	0.932–1.389	0.206	0.979	0.848–1.131	0.772
Hypertension	1.815	0.555–5.931	0.324	1.298	0.525–3.207	0.572
Diabetes Mellitus	1.333	0.343–5.178	0.678	0.700	0.226–2.166	0.536
MetS	1.818	0.483–6.850	0.377	1.300	0.492–3.434	0.597
Total-PSA (ng/ml)	1.013	0.964–1.064	0.618	1.004	0.986–1.023	0.645
Free-PSA (ng/ml)	1.180	0.737–1.888	0.491	1.249	0.884–1.765	0.208
PSA						
PSA ≤ 4	1.286	0.213–7.760	0.784	NA	NA	NA
PSA > 4	Ref	Ref	Ref	Ref	Ref	Ref
Free PSA/Total PSA ratio						
≤ 18%	Ref	Ref	Ref	Ref	Ref	Ref
> 18%	1.462	0.372–5.751	0.586	0.581	0.090–3.738	0.567
PV (ml)	1.001	0.986–1.017	0.904	0.991	0.978–1.005	0.192
PI-RADS						
≤ 3	0.524	0.167–1.638	0.266	1.136	0.437–2.957	0.793
> 3	Ref	Ref	Ref	Ref	Ref	Ref
Number of biopsy cores	1.295	0.372–4.515	0.685	0.942	0.824–1.077	0.381
Rate of positive cores in biopsy	5.455	0.126–236.209	0.378	8.214	0.837–80.569	0.071
Clinical stage						
T1	Ref	Ref	Ref	Ref	Ref	Ref
T2	3.375	0.283–40.254	0.336	2.200	0.190–25.516	0.528
T3	4.000	0.211–75.659	0.355	5.333	0.343–82.827	0.232
Triglyceride	0.955	0.684–1.333	0.786	1.082	0.639–1.832	0.770
HDL-C	1.446	0.216–9.666	0.704	7.735	0.998–59.957	**0.050**
Lymphocyte counts	0.789	0.359–1.736	0.557	1.261	0.703–2.262	0.437
Percentage of lymphocyte (%)	0.951	0.904–1.000	**0.052**	0.995	0.959–1.032	0.780
Neutrophile counts	1.326	1.005–1.748	**0.046**	1.114	0.967–1.283	0.134
Percentage of neutrophile	1.022	0.985–1.059	0.248	0.989	0.962–1.017	0.450
NLR	1.128	0.980–1.297	0.092	1.013	0.972–1.056	0.532
Hb	0.964	0.923–1.007	0.101	0.983	0.956–1.012	0.248

Significant value was in bold

*GSU* Gleason score upgrading, *PSA* prostate-specific antigen, *PV* prostate volume, *PI-RADS* prostate imaging reporting and data system, *HDL-C* high-density lipoprotein cholesterol, *Hb* hemoglobin, *ISUP* International Society of Urological Pathology

It's worth mentioning that PI-RADS ≤ 3 has the opposite on GSU in two subgroups. In the GS = 6 group, PI-RADS ≤ 3 had a lower risk of GSU (OR: 0.524, 95% CI: 0.167–1.638), and PI-RADS ≤ 3 had a higher risk of GSU (OR: 1.136, 95% CI: 0.437–2.957) in GS = 7 group although no significances displayed in either group.

## Discussion

Our study showed that inflammatory cell numbers and high HDL levels were GSU risk factors. A high percentage of lymphocyte and neutrophile count was highly correlated with GSU in patients with GS = 6, whereas a high serum HDL level was associated with GSU in patients with GS = 7. Laboratory examination data may provide urologists with more information on predicting GSU besides currently wide-used biopsy and image evaluations when planning therapeutic protocols.

The current study showed that a high serum HDL level is a risk factor for GSU in patients diagnosed with high-grade prostate cancer (GS = 7). Studies have shown that patients with high-grade prostate cancer also tended to exhibit high HDL levels [9]. High serum HDL levels were associated with an increased risk of high-grade and overall prostate cancer diagnosis [10, 11]. The relation between HDL and prostate cancer suggests that HDL may involve cell proliferation and disease progression of prostate cancer. It is underlined by a recent in vivo study that significantly higher HDL levels and larger tumors were observed in WT mice compared to in mice knockout of scavenger receptor class B type 1 (SR-B1), a receptor that mediates HDL uptake into cells [15]. Our findings further showed a high serum HDL level was associated with GSU in patients diagnosed with high-grade prostate cancer. This supports the HDL-prostate cancer link and provides urologists with a convenient and inexpensive tool to get more information about therapeutic protocols.

Our study also proposed that an elevated neutrophil count and a reduced percentage of lymphocytes were risk factors of GSU in patients diagnosed with low-risk prostate cancer (GS = 6). However, whether inflammatory factors could predict GSU in either low- or high-grade prostate cancer remains conflicting [13, 16]. The human immune system has different responses in different stages of tumorigenesis. A hypothesis could illustrate our findings that lymphocytes were recruited in the early stage of prostate cancer [17], and an increase in neutrophils is needed in the following advanced stage.

In current results, although the PSA value was elevated as the severity of diagnosed prostate cancer increased, no significant difference was displayed between GSU and NGSU groups regardless of grades of prostate cancer. Many studies consider PSA an independent predictor of GSU [7, 8, 18, 19]. However, an increased PSA is not triggered by prostate cancer only, therefore could not represent the whole picture. Colleselli et al. [17] reported that patients with the PSA of 2–3.9 and 4–10 ng/mL were upgraded by 32.6% and 44%, respectively. Santok et al. [18] GS = 6 patients with a PSA level of 10–20 ng/mL had an increased risk of GSU to GS≧8. Given that the reported PSA level predicting GSU ranges from 7 to 10 ng/mL [7, 8, 17], the broad range suggests different inclusion criteria, study design, and study cohort may result in different conclusions. Therefore, the clinic must identify relevant laboratory examination data for GSU prediction.

Recently metabolic syndrome has been associated with an increased risk of advanced disease [19]. Their study investigated the association of metabolic syndrome with the risk of prostate cancer upgrading and upstaging after radical prostatectomy. Patients with metabolic syndrome presented the worst accuracy and kappa coefficient of agreement between needle biopsy and radical prostatectomy specimens. Therefore, results should be evaluated carefully in patients with metabolic syndrome.

To improve the histologic grading system for scoring the invasiveness of prostate cancer, a study indicated that prostate-specific antigen density is a valuable predictor of upgrading and upstaging in men with prostate cancer who were candidates for surgery and is accurate in selecting patients for active surveillance [20]. Another study demonstrated that the new Epstein Gleason score classification significantly reduces upgrading in prostate cancer patients [21]. Their results showed that the five-tier Gleason grading system presented a lower clinically significant upgrading rate and a similar clinically significant downgrading rate compared to the 2005 ISUP classification. When evaluating their accuracy, the new five-tier Gleason grading system presented a better specificity and a better negative predictive value. Together, these results indicated that prostate-specific antigen density and a five-tier Gleason grading system could improve the inaccuracy of GSU.

There are two limitations of this research. First, it is a single-center study with a small sample size, which could cause selection bias. Second, in a retrospective study, we could not include other known factors of inflammation and cholesterol because they are not routinely included in laboratory examinations for prostate cancer. Neutrophile count, percentage of lymphocyte, and serum HDL level are inexpensive and easily performed laboratory examination data. Extensive sample size studies are needed to clarify the predictive roles in GSU.

## Conclusions

For patients diagnosed with low-grade prostate cancer (GS = 6), high neutrophile count and low percentage of lymphocytes may correlate with GSU; for those with high-grade prostate cancer (GS = 7), high HDL may connect with GSU. These laboratory examination data may provide urologists with more information before making a therapeutic protocol.

## Data Availability

The datasets generated during the current study are published in this article.

## Abbreviations

GSU: Gleason score upgrading
GS: Gleason score
HDL: High-density lipoprotein
PV: Prostate volume
PSA: Prostate-specific antigen
OR: Odds ratio
Cis: Confidence intervals
